# Draft genome sequence of the basidiomycetous fungus *Tinctoporellus epimiltinus* strain RS1

**DOI:** 10.1016/j.dib.2019.103796

**Published:** 2019-02-28

**Authors:** Ranjita Subramaniam, Shafiquzzaman Siddiquee, Kennedy Aaron Aguol, Mohammad Zahirul Hoque, Subbiah Vijay Kumar

**Affiliations:** a Biotechnology Research Institute, Universiti Malaysia Sabah, Jalan UMS, 88400 Kota Kinabalu, Sabah, Malaysia; b Centre of the Promotion of Knowledge and Language Learning, Universiti Malaysia Sabah, Jalan UMS, 88400 Kota Kinabalu, Sabah, Malaysia; c Department of Pathobiology and Medical Diagnostics, Faculty of Medicine and Health Sciences, Universiti Malaysia Sabah, Jalan UMS, 88400 Kota Kinabalu, Sabah, Malaysia

## Abstract

Members of the genus *Tinctoporellus*, which belong to the wood-degrading basidiomycetes, possess the ability to synthesize an array of industrially potent enzymes and metabolites. Here, we present the draft genome sequence of the species *Tinctoporellus epimiltinus* strain RS1, which is the first to represent its genus. The genome was sequenced using Illumina's 2 × 150 bp paired-end Nextera protocol. The draft genome assembly was 46.2 Mb in size consisting of 13,791 protein coding genes. Identification of carbohydrate active enzymes and laccases from the data may be useful in order to harness the metabolic potentials of the fungi. The data can be accessed at ENA under the accession number FTLJ00000000.

**Table:** Specifications table

Subject area	Biology
More specific subject area	Mycology, Genomics
Type of data	Genomic sequence, gene prediction and annotation of *Tinctoporellus epimiltinus* strain RS1
How data was acquired	Whole genome was sequenced with an Illumina MiSeq System
Data format	Raw sequencing reads, draft genome assembly and gene prediction
Experimental factors	*Tinctoporellus epimiltinus* RS1 was identified from a mixed culture plate growing together with colonies of *Trichoderma* spp. Fungal colony purification was performed using serial dilution. DNA was isolated using CTAB and 16S rDNA was performed to confirm species identity.
Experimental features	The genome was assembled with CLC Genomic Workbench v6.5.1. Gene prediction and annotation was performed with GeneMark-ES version2.3.
Data source location	Strain *RS1* was found in a PDA culture plate growing together with colonies of *Trichoderma* spp. The fungi were obtained from soil samples that were originally collected from an oil palm plantation in Sabah (North Borneo), Malaysia.
Data accessibility	Data has been deposited at DDBJ/ENA/GenBank under the accession number FTLJ00000000. The version described in this paper is the first version, FTLJ01000000.
Related research article	R.P. Kancherla, M.B. Durling, J. Stenlid, N. Hőgberg, Draft genome of the brown-rot fungus Fomitopsis pinicola GR9-4. Data in Brief 15 (2017) 496–500 [1]

**Table:** 

**Value of the data** • The first draft genome of *Tinctoporellus epimiltinus* RS1 • The first genome under the genus *Tinctoporellus* to be sequenced. • The fungi possess the ability to produce degrading enzymes and useful metabolites. • The draft genome will accelerate functional genomics research and help to understand the genetic make-up of economically important genes.

## 1 Data

The data presented here represents the genome sequencing, assembly, and annotation of the lignin degrading fungal species *Tinctoporellus epimiltinus* RS1. Illumina sequencing data generated 29.22 million paired-end reads with a total output of 4.0 Gb. After quality trimming at Q>30, approximately 92.44% of the reads were assembled into the nuclear genome consisting of 2,002 scaffolds larger than 1,000 bp in size. The N50 contig length was 58.9 Kb with an average coverage of 74×. The resulting draft genome was 46,175,157 bp in size with a G+C content of 57.54%. Gene prediction analysis using GeneMark-ES version2.3 resulted in 13,791 protein coding genes. The draft genome assembly information of *Tinctoporellus epimiltinus* agrees well with other sequenced fungal genomes [1], [2].

**Fig. 1:**
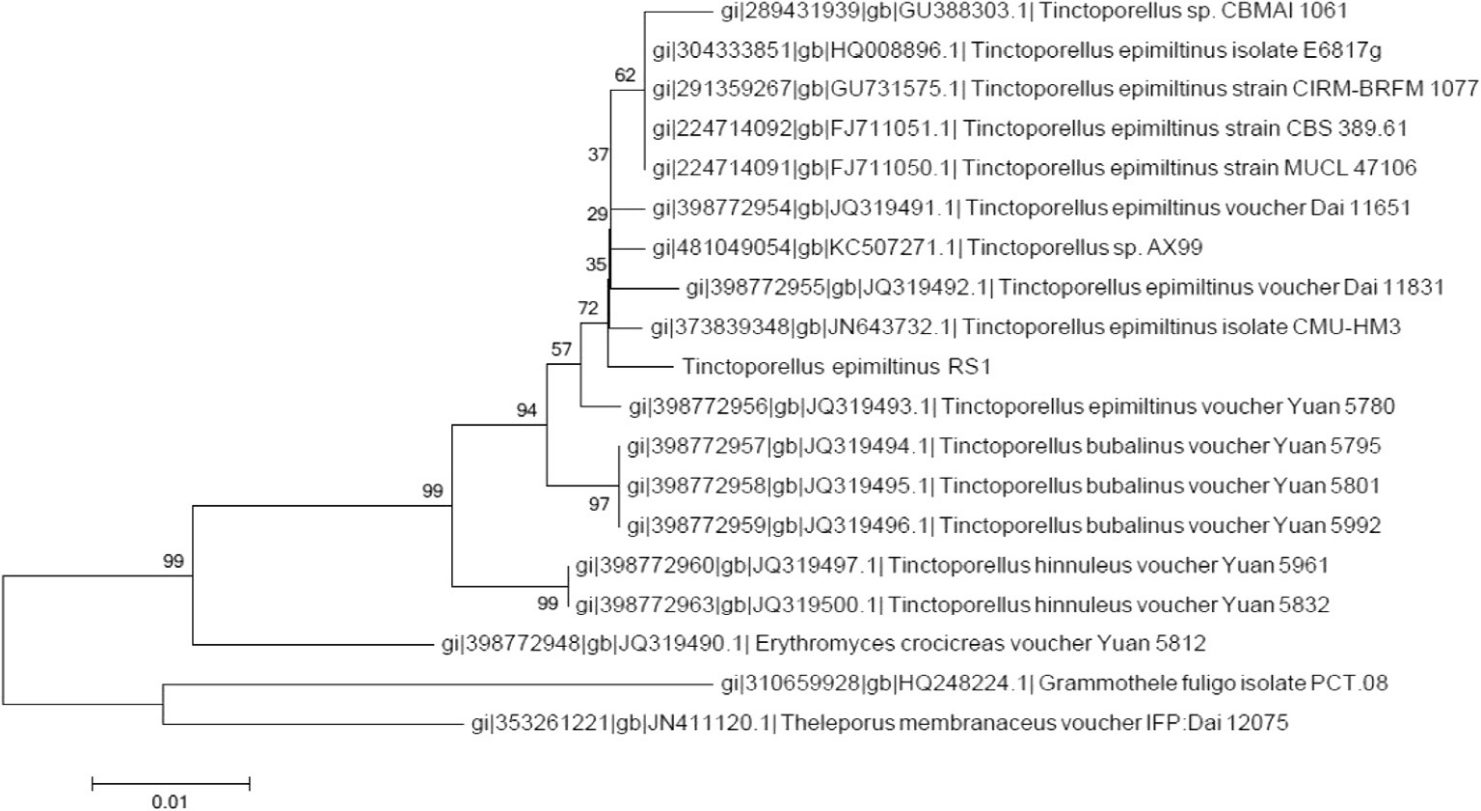
Phylogenetic tree based on the Neighbor-Joining method, Kimura 2-parameter with 1000 bootstrap replicates. Strain RS1 is grouped together with other *T. epimiltinus* isolates.

Ninety-one percent of the predicted genes were annotated based on BLASTp similarity searches against a selection of the nr database (Fungi) with an e-value of 10^−3^. The data contains 861 secreted protein candidates. The secretome data of the draft genome contains 259 genes coding for different carbohydrate-active enzymes (CAZymes), with 123 glycoside hydrolases, 51 carbohydrate esterases and 64 with auxiliary activities, among them. The data includes 12 genes encoding for manganese peroxidases (MnPs) and 14 genes encoding laccases, among the enzymes with auxiliary activities. This whole-genome shotgun project has been deposited at DDBJ/ENA/GenBank under the accession number FTLJ00000000. The version described in this paper is the first version, FTLJ01000000. An internal transcribed spacer (ITS)-region phylogenetic tree based on Neighbour-Joining method places strain RS1 with other *T. epimiltinus* species (Fig. 1).

## 2 Experimental design, materials and methods

### 2.1 Genomic DNA extraction and sequencing

The fungal sample designated as *Tinctoporellus epimiltinus* RS1 was recovered into pure culture when found growing in a mixed culture plate together with colonies of *Trichoderma* spp. These were originally collected from soil samples from an oil palm plantation in Sabah (North Borneo), Malaysia. Fungal colony purification was performed based on serial dilution technique complemented with pour plate method as described by Emoghene et al. [3]. DNA isolation was then performed by using the CTAB method with modification [4]. Subsequently, species identification was carried out using macro- and microscopic analysis. We also sequenced the internal transcribed spacer (ITS)-region after PCR amplification using the respective ITS 1 (5′-TCCGTAGGTGAACCTGCGG-3′) and ITS 4 (5′-TCCTCCGCTTATTGATATGC-3′) forward and reverse primers. In addition, the genomic DNA was converted into sequencing-ready library using the Nextera DNA Sample Preparation Kit (Illumina, San Diego, CA). The library was then sequenced on the Illumina MiSeq (150-bp paired-end reads) platform.

### 2.2 Genome assembly and annotation

*De novo* assembly was carried out using the CLC Genomic Workbench version 6.5.1. Quality trimming was performed at Q>30 and the resulting reads were assembled into scaffolds. The self-training GeneMark-ES software [5] was used to predict protein coding sequences. Predicted proteins were classified as secreted when predicted to have a signal peptide using SignalP version 4.1 [6], to have no transmembrane domains according to TMHMM version 2.0 [7], and to have no GPI anchors according to BIG-PI fungal predictor [8]. Secretome analysis was performed using dbCAN version 6.0 [9] following a similar approach taken to sequence the brown-rot fungus *Fomitopsis pinicola* [1]. The output was then blast against the protein database using MolQuest for lignin peroxidases, manganese peroxidases, laccases, versatile peroxidases and DyP-like protein sequences. GeneMark-ES predictions were compared with Fgenesh [10] and Augustus [11] which was pre-trained with the gene model of *Phanerochaete chrysosporium* to determine exon/intron boundary of the genes.
